# Lysyl oxidase-like 2 as a predictor of hepatocellular carcinoma in patients with hepatitis C virus after sustained virological response

**DOI:** 10.1038/s41598-024-61366-y

**Published:** 2024-05-13

**Authors:** Takeshi Chida, Kazuyoshi Ohta, Hidenao Noritake, Masahiro Matsushita, Gou Murohisa, Fujito Kageyama, Yuzo Sasada, Tatsuki Oyaizu, Minoru Tsugiki, Katsutoshi Tamakoshi, Takeyuki Nakajima, Takafumi Suda, Kazuhito Kawata

**Affiliations:** 1 Second Department of Internal Medicine, Hamamatsu University School of Medicine, 1-20-1 Handayama, Hamamatsu, Shizuoka 431-3192 Japan; 2 Department of Regional Medical Care Support, Hamamatsu University School of Medicine, 1-20-1 Handayama, Hamamatsu, Shizuoka 431-3192 Japan; 3 Department of Gastroenterology, Shimada General Medical Center, 1200-5 Noda, Shimada, Shizuoka 427-8502 Japan; 4 Department of Hepatology, Seirei Hamamatsu General Hospital, 2-12-12 Sumiyoshi, Hamamatsu, Shizuoka 430-8558 Japan; 5 Department of Hepatology, Hamamatsu Medical Center, 328 Tomitsuka-Cho, Hamamatsu, Shizuoka 432-8580 Japan; 6 Department of Hepatology, Iwata City Hospital, 512-3 Ookubo, Iwata, Shizuoka 438-8550 Japan; 7 Department of Gastroenterology, Shizuoka City Shizuoka Hospital, 10-93 Otemachi, Shizuoka, Shizuoka 420-8630 Japan; 8 Minoru Medical Clinic, 1784-1 Mishima-Cho, Hamamatsu, Shizuoka 430-0853 Japan; 9 Tamakoshi Clinic, 262-1 Maruzuka-Cho, Hamamatsu, Shizuoka 435-0046 Japan; 10 Elm Medical Clinic, 5-17-22 Handayama, Hamamatsu, Shizuoka 431-3125 Japan

**Keywords:** Lysyl oxidase-like 2 (LOXL2), Hepatocellular carcinoma (HCC), Hepatitis C virus (HCV), Sustained virological response (SVR), Alpha-fetoprotein (AFP), Liver diseases, Predictive markers

## Abstract

Lysyl oxidase-like 2 (LOXL2) mediates the crosslinking of extracellular collagen, reflecting qualitative changes in liver fibrosis. This study aimed to validate the utility of serum LOXL2 levels as a predictive biomarker for the development of hepatocellular carcinoma (HCC) in patients with hepatitis C virus (HCV) infection who achieved a sustained virological response (SVR). This retrospective study included 137 patients with chronic HCV infection without history of HCC development and who achieved SVR via direct-acting antiviral therapy. Median LOXL2 levels decreased significantly after SVR achievement (pre-Tx, 2.33 ng/mL; post-Tx, 1.31 ng/mL, *p* < 0.001). Post-Tx LOXL2 levels, fibrosis-4 index, platelet counts, Wisteria floribunda agglutinin-positive human Mac-2 binding protein levels, and alpha-fetoprotein (AFP) levels were identified as independent predictive factors for post-SVR HCC development in the univariate analysis. The incidence of post-SVR HCC development was significantly higher in patients with post-Tx LOXL2 levels ≥ 2.08 ng/mL and AFP levels ≥ 5.0 ng/mL than in patients with elevated levels of either marker or with lower marker levels. Serum LOXL2 levels can serve as a predictive biomarker for HCC development after achieving SVR. The combination of serum LOXL2 and AFP levels provides robust risk stratification for HCC development after SVR, suggesting an enhanced surveillance strategy.

## Introduction

Hepatitis C virus (HCV) infection is a global health concern, affecting more than 58 million people worldwide, and a major cause of chronic hepatitis, cirrhosis, and hepatocellular carcinoma (HCC)^1^. With the development of direct-acting antiviral (DAA) therapy, HCV treatment has made remarkable progress, achieving sustained virological response (SVR) rates of over 95% across all genotypes^2,3^. Although treatment with DAAs reduces the risk of HCC development and liver disease-related mortality associated with HCV infection, the risk of HCC development after SVR remains between 0.9 and 2.96 per 100 patient-years^4–6^, indicating that HCC still occurs in some patients. As the number of patients who achieve SVR increases, the importance of accurate prediction and appropriate surveillance for HCC after SVR becomes significant.

Several risk factors for HCC development after SVR have been reported^7–11^, of which liver fibrosis is a significant predictor. Although liver biopsy is considered the gold standard for fibrosis assessment, it has certain limitations, including sampling variability and the potential for severe complications^12^. Consequently, the demand for non-invasive biomarkers for the evaluation of liver fibrosis and prediction of HCC development after SVR has grown^13^.

Lysyl oxidase-like 2 (LOXL2) is an enzyme that catalyzes collagen crosslinking in the extracellular matrix (ECM)^14,15^. The liver collagens modified by LOXL2 become tightly crosslinked and difficult to solubilize. The LOX family consists of LOX and LOXL1-4; LOXL2 is expressed in the liver and other organs, including the breast, lungs, gastrointestinal tract, and kidneys, where it has various functions^16^. It is associated with various pathogenesis-related processes, such as post-translational modification of ECM collagens, epithelial-mesenchymal transition (EMT), angiogenesis, and cancer progression or metastasis^17–21^.

In general, liver fibrosis, from chronic hepatitis to early-stage cirrhosis, ameliorates to some extent after achieving SVR with interferon (IFN) or DAA^22–27^ treatment; however, in some cases where insoluble fibers are already deposited, fibrosis is highly resistant to fibrinolysis, and significant improvement is not achieved after SVR, thus increasing the risk for HCC^28,29^. Markers such as LOXL2 that specifically focus on the "quality" or "solubility", rather than the severity, of fibrosis can also be valuable for predicting HCC after achieving SVR.

Considering cost-effectiveness, the modality and frequency of HCC screening should be individually optimized according to personal risk profiles^9,30^. Establishing a valid HCC risk assessment method using non-invasive biomarkers to provide a personalized follow-up system is essential. The aim of this study was to verify the utility of LOXL2 as a predictive biomarker for HCC development after SVR in patients with HCV, and to use it to stratify risk, enabling an efficient surveillance strategy.

## Results

### Patient characteristics

The baseline characteristics of the 137 patients are summarized in Table 1. The median age was 67 years, 73 (53.3%) patients were male, 65 (47.4%) had a history of an IFN-based regimen, 6 (4.4%) consumed 20 g or more of alcohol per day, and 22 (16.1%) had diabetes mellitus. The median observation period was 4.8 years (0.2–7.3). The median alpha-fetoprotein (AFP) and LOXL2 levels in the serum were significantly higher in the pre-treatment (pre-Tx) than in the post-treatmant (post-Tx) samples (AFP; 5.8 and 3.5 ng/mL, LOXL2; 2.33 and 1.31 ng/mL, respectively). The median LOXL2 level in the patients with HCV was significantly higher than that in healthy volunteers (N = 13) (2.33 vs. 1.39 ng/mL, *p* = 0.002; Supplementary Fig. 1).

**Table 1:** Characteristics of the patients enrolled in the present study.

Factors	Value	Pre-Tx	Post-Tx	*p* value
Patients, n	137			
Age (year)	67 (32–84)			
Sex (male, n (%))	73 (53.3)			
BMI (kg/m^2^)	22.0 (15.7–34.1)			
Alcohol intake (> = 20 g/day, n(%))	6 (4.4)			
Diabetes mellitus, n (%)	22 (16.1)			
Past history of IFN treatment, n (%)	65 (47.4)			
HCV serogroup1, n (%)	137 (100)			
DAA regimen, n (%)				
DCV/ASV	83 (60.6)			
SOF/LDV	54 (39.4)			
Pre-Tx HCV titer, (Log_10_ IU/mL)	6.0 (2.8–7.2)			
Observation period (year)	4.8 (0.2–7.3)			
Platelet counts (× 10^9^/L)		154 (46–480)	159 (53–458)	0.010
AST (U/L)		41 (16–217)	22 (12–47)	< 0.001
ALT (U/L)		41 (10–233)	15 (3–59)	< 0.001
Total bilirubin (mg/dL)		0.7 (0.3–2.0)	0.8 (0.3–1.9)	0.005
GGT (U/L)		31 (13–225)	20 (10–105)	< 0.001
Albumin (g/dL)		4.1 (2.9–5.1)	4.3 (3.1–5.2)	< 0.001
AFP (ng/mL)		5.8 (1.0–158.0)	3.5 (1.0–33.0)	< 0.001
WFA^+^-M2BP (COI)		2.66 (0.42–20.0)	1.06 (0.27–11.12)	< 0.001
FIB-4 index		2.92 (0.54–14.66)	2.55 (0.54–9.38)	< 0.001
ALBI		− 2.79 (− 3.65 to − 1.50)	− 2.96 (− 3.75 to − 1.71)	< 0.001
LOXL2 (ng/mL)		2.33 (0.51–4.60)	1.31 (0.18–3.16)	< 0.001

Data are presented as median (range) or numbers and percentages if indicated. The Wilcoxon signed-rank test was used to assess differences between the two paired groups. Abbreviations: AFP, alpha-fetoprotein; ALBI, albumin-bilirubin; ALT, alanine aminotransferase; AST, aspartate aminotransferase; ASV, asunaprevir; BMI, body mass index; COI, cut-off index; DAA, direct-acting antiviral; DCV, daclatasvir; FIB‐4, fibrosis‐4; GGT, gamma-glutamyl transpeptidase; HCV, hepatitis C virus; IFN, interferon; LDV, ledipasvir; LOXL2, lysyl oxidase-like 2; pre-Tx, pre-treatment; post-Tx, post-treatment; SOF, sofosbuvir; SVR, sustained virological response; WFA^+^-M2BP, Wisteria floribunda agglutinin-positive human Mac-2 binding protein.

### Correlation of serum LOXL2 levels with liver fibrosis or tumor markers

We investigated whether serum LOXL2 levels correlated with various markers for liver fibrosis or tumor. Pre- and post-Tx LOXL2 levels did not show strong correlations with platelet counts, Wisteria floribunda agglutinin-positive human Mac-2 binding protein (WFA^+^-M2BP) levels, fibrosis-4 (FIB-4) index, albumin-bilirubin (ALBI), or AFP levels in the serum (Supplementary Fig. 2).

### HCC development in patients who achieved SVR

**Figure 1:**
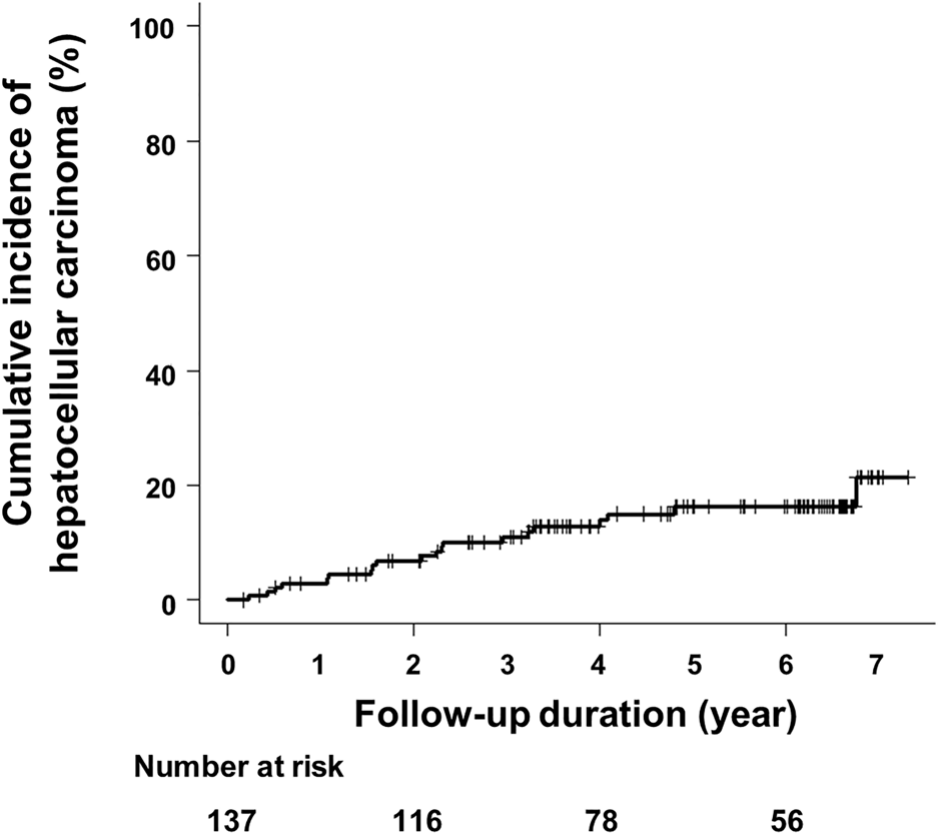
Cumulative Incidence of HCC after Achieving SVR with DAA Therapy. The cumulative incidence of HCC development after SVR was assessed using the Kaplan–Meier method. Abbreviations: DAA, direct-acting antiviral; HCC, hepatocellular carcinoma; SVR, sustained virological response.

During the observation period, 20 of the 137 patients (14.6%) developed post-SVR HCC. Detail backgrounds of patients developed post-SVR HCC were shown in Supplementary Table 1. The cumulative post-SVR incidence of HCC at 1, 3, and 5 years was 3.0, 11.0, and 16.3%, respectively (Fig. 1). Patients with post-SVR HCC were significantly older (*p* = 0.034) and had lower platelet counts (*p* = 0.032), higher AFP levels (*p* < 0.001), higher WFA^+^-M2BP levels (*p* = 0.047), and higher FIB-4 index (*p* = 0.012) post-Tx than those who did not develop post-SVR HCC. Pre- and post-Tx serum LOXL2 levels in the patients with post-SVR HCC were also higher than in those without, but the difference was not statistically significant (Table 2).

**Table 2:** Characteristics of patients with or without HCC development after SVR.

Factors	non-HCC	HCC	*p-value*
Patients, n	117	20	
Age (year)	66 (32–84)	71 (43–82)	0.034
Sex (male, n (%))	61 (52.1)	12 (60.0)	0.683
BMI (kg/m^2^)	22.0 (15.7–34.1)	22.1 (16.8–31.6)	0.323
Alcohol intake (g/day)	5 (4.3)	1 (5.0)	1.000
Diabetes mellitus, n (%)	16 (13.7)	6 (30.0)	0.132
Past history of IFN treatment, n (%)	54 (46.2)	11 (55.0)	0.624
Platelet counts (× 10^9^/L)			
At Pre-Tx	157 (46–480)	122 (59–201)	0.020
At Post-Tx	160 (53–458)	115 (65–221)	0.032
AST (U/L)			
At Pre-Tx	41 (16–217)	43 (25–146)	0.392
At Post-Tx	22 (12–47)	25 (16–36)	0.089
ALT (U/L)			
At Pre-Tx	41 (10–184)	41 (19–233)	0.903
At Post-Tx	14 (3–59)	16 (5–50)	0.302
AFP (ng/mL)			
At Pre-Tx	5.0 (1.0–182)	11.2 (4.0–45.2)	< 0.001
At Post-Tx	3.0 (1.0–9.7)	5.7 (2.0–33.0)	< 0.001
WFA^+^-M2BP (COI)			
At Pre-Tx	2.42 (0.42–20.0)	3.71 (1.40–13.75)	0.030
At Post-Tx	1.04 (0.27–11.12)	1.52 (0.39–4.92)	0.047
FIB-4 index			
At Pre-Tx	2.60 (0.54–14.66)	4.09 (2.17–10.65)	0.012
At Post-Tx	2.50 (0.54–9.38)	3.71 (0.89–7.95)	0.012
ALBI			
At Pre-Tx	− 2.84 (-3.65 to -1.50)	− 2.65 (− 3.58 to − 1.86)	0.011
At Post-Tx	− 2.99 (− 3.75 to − 1.71)	− 2.81 (− 3.50 to -2.02)	0.063
LOXL2 (ng/mL)			
At Pre-Tx	2.22 (0.51–4.60)	2.53 (0.58–3.86)	0.342
At Post-Tx	1.28 (0.18–3.16)	1.74 (0.40–2.71)	0.124

Data are presented as median (range) or numbers and percentages if indicated. The chi-square and Mann–Whitney U tests were used to evaluate categorical data and continuous variables, respectively. Abbreviations: AFP, alpha-fetoprotein; ALBI, albumin-bilirubin; ALT, alanine aminotransferase; AST, aspartate aminotransferase; BMI, body mass index; COI, cut-off index; FIB‐4, fibrosis‐4; HCC, hepatocellular carcinoma; IFN, interferon; LOXL2, lysyl oxidase-like 2; pre-Tx, pre-treatment; post-Tx, post-treatment; SVR, sustained virological response; WFA^+^-M2BP, Wisteria floribunda agglutinin-positive human Mac-2 binding protein.

### Predictive factors associated with HCC development after achieving SVR

Univariate analysis identified factors that predicted the risk for HCC development after achieving SVR. Cox regression analysis was performed on six backgrounds (age, sex, body mass index [BMI], alcohol intake, presence of diabetes mellitus, and history of IFN treatment) and eight pre- or post-Tx variables (platelet counts, aspartate aminotransferase [AST], alanine aminotransferase [ALT], AFP, and WFA^+^-M2BP levels, FIB-4 index, ALBI, and LOXL2 levels). The cut-off values for these factors, except ALBI and LOXL2, were set according to previous reports^7,31–39^. The cut-off values for ALBI and LOXL2 were set as − 2.27, which distinguishes a modified (m)ALBI grade 2a from 2b^40–42^, and 2.08, based on a receiver operating characteristic (ROC) curve, respectively (Supplementary Fig. 3).

Among background factors, age ≥ 65 years (hazard ratio [HR] 3.117, 95% confidence interval [CI] = 1.190–8.164, *p* = 0.021) and diagnosis with diabetes mellitus (HR 2.624, 95% CI = 1.007–6.840, *p* = 0.048) were identified as risk factors for post-SVR HCC development. Regarding pre-Tx factors, platelet count < 120 × 10^9^/L (HR 2.453, 95% CI = 1.019–5.910, *p* = 0.045), AFP level ≥ 10.0 ng/mL (HR 3.081, 95% CI = 1.268–7.484, *p* = 0.013), WFA^+^-M2BP level ≥ 2.8 (cut-off index, COI) (HR 3.138, 95% CI = 1.204–8.181, *p* = 0.019), and FIB-4 index ≥ 3.25 (HR 3.604, 95% CI = 1.382–9.400, *p* = 0.009) were identified. Platelet count < 120 × 10^9^/L (HR 3.150, 95% CI = 1.302–7.622, *p* = 0.011), AFP level ≥ 5.0 ng/mL (HR 4.486, 95% CI = 1.781–11.30, *p* = 0.001), WFA^+^-M2BP level ≥ 1.0 COI (HR 3.383, 95% CI = 1.130–10.13, *p* = 0.029), FIB-4 index ≥ 2.9 (HR 2.703, 95% CI = 1.077–6.783, *p* = 0.034), and LOXL2 level ≥ 2.08 ng/mL (HR 2.939, 95% CI = 1.203–7.178, *p* = 0.018) were identified among post-Tx factors (Table 3).

**Table 3:** Factors Associated with HCC Development after SVR.

Factors	Category	HR (95% CI)	*p-value*
Age (year)	≥ 65	3.117 (1.190–8.164)	0.021
Sex	Male		
BMI (kg/m^2^)	≥ 23.5		
Alcohol intake (g/day)	≥ 20		
Diabetes mellitus	Yes	2.624 (1.007–6.840)	0.048
Past history of IFN treatment	Yes		
Platelet counts (× 10^9^/L)			
At Pre-Tx	< 120	2.453 (1.019–5.910)	0.045
At Post-Tx	< 120	3.150 (1.302–7.622)	0.011
AST (U/L)			
At Pre-Tx	≥ 40		
At Post-Tx	≥ 40		
ALT (U/L)			
At Pre-Tx	≥ 40		
At Post-Tx	≥ 40		
AFP (ng/mL)			
At Pre-Tx	≥ 10	3.081 (1.268–7.484)	0.013
At Post-Tx	≥ 5	4.486 (1.781–11.30)	0.001
WFA^+^-M2BP (COI)			
At Pre-Tx	≥ 2.8	3.138 (1.204–8.181)	0.019
At Post-Tx	≥ 1.0	3.383 (1.130–10.13)	0.029
FIB-4 index			
At Pre-Tx	≥ 3.25	3.604 (1.382–9.400)	0.009
At Post-Tx	≥ 2.90	2.703 (1.077–6.783)	0.034
ALBI			
At Pre-Tx	> -2.27		
At Post-Tx	> -2.27		
LOXL2 (ng/mL)			
At Pre-Tx	≥ 2.50		
At Post-Tx	≥ 2.08	2.939 (1.203–7.178)	0.018

Statistical analysis was performed using the Cox proportional hazards model. AFP, alpha-fetoprotein; ALBI, albumin-bilirubin; ALT, alanine aminotransferase; AST, aspartate aminotransferase; BMI, body mass index; CI, confidence interval; FIB‐4, fibrosis‐4; HCC, hepatocellular carcinoma; HR, hazard ratio; IFN, interferon; LOXL2, lysyl oxidase-like 2; pre-Tx, pre-treatment; post-Tx, post-treatment; SVR, sustained virological response; WFA^+^-M2BP, Wisteria floribunda agglutinin-positive human Mac-2 binding protein.

Multivariate analysis was performed to investigate confounding factors between serum LOXL2 levels and other factors. Combinations of serum LOXL2 levels with serum AFP level, platelet count, age, and presence of diabetes mellitus, were identified as independent risk factors for post-SVR HCC development (Table 4). In contrast, when serum LOXL2 levels were combined with the FIB-4 index or WFA^+^-M2BP levels, only LOXL2 levels were extracted.

**Table 4:** Factors Associated with Development of HCC after SVR (Multivariate Analysis).

Variables	Category	Model 1		Model 2		Model 3		Model 4		Model 5		Model 6	
		HR (95% CI)	*p* value	HR (95% CI)	*p* value	HR (95% CI)	*p* value	HR (95% CI)	*p* value	HR (95% CI)	*p* value	HR (95% CI)	*p* value
Post-Tx LOXL2 (ng/mL)	≥ 2.08	3.239 (1.332–7.876)	0.010	2.542 (1.032–6.257)	0.042	2.502 (1.019–6.143)	0.045	2.645 (1.077–6.496)	0.034	2.662 (1.084–6.538)	0.033	2.926 (1.201–7.127)	0.018
Post-Tx AFP (ng/mL)	≥ 5	4.796 (1.904–12.08)	< 0.001										
Post-Tx platelet counts (× 10^9^/L)	< 120			2.802 (1.148–6.838)	0.024								
Post-Tx WFA^+^-M2BP (COI)	≥ 1.0					2.965 (0.981–8.965)	0.054						
Post-Tx FIB-4 index	≥ 2.9							2.451 (0.971–6.185)	0.058				
Age (years)	≥ 65									2.862 (1.090–7.517)	0.033		
Diabetes mellitus	Yes											2.620 (1.006–6.824)	0.049

Statistical analysis was performed using the Cox proportional hazards model. Abbreviations: AFP, alpha-fetoprotein; CI, confidence interval; COI, cut-off index; FIB-4, fibrosis-4; HCC, hepatocellular carcinoma; HR, hazard ratio; LOXL2, lysyl oxidase-like 2; post-Tx, post-treatment; SVR, sustained virological response; WFA^+^-M2BP, Wisteria floribunda agglutinin-positive human Mac-2 binding protein.

### Stratification for the risk of HCC development after achieving SVR with predictive factors

The cumulative post-SVR incidence rate of HCC was examined using the Kaplan–Meier method. Significant differences in the post-SVR incidence rate of HCC were observed when the patients were divided into two groups by either LOXL2 (< 2.08 vs. ≥ 2.08 ng/mL) or AFP (< 5.0 vs. ≥ 5.0 ng/mL) post-Tx levels (*p* = 0.013 and < 0.001, respectively).

**Figure 2:**
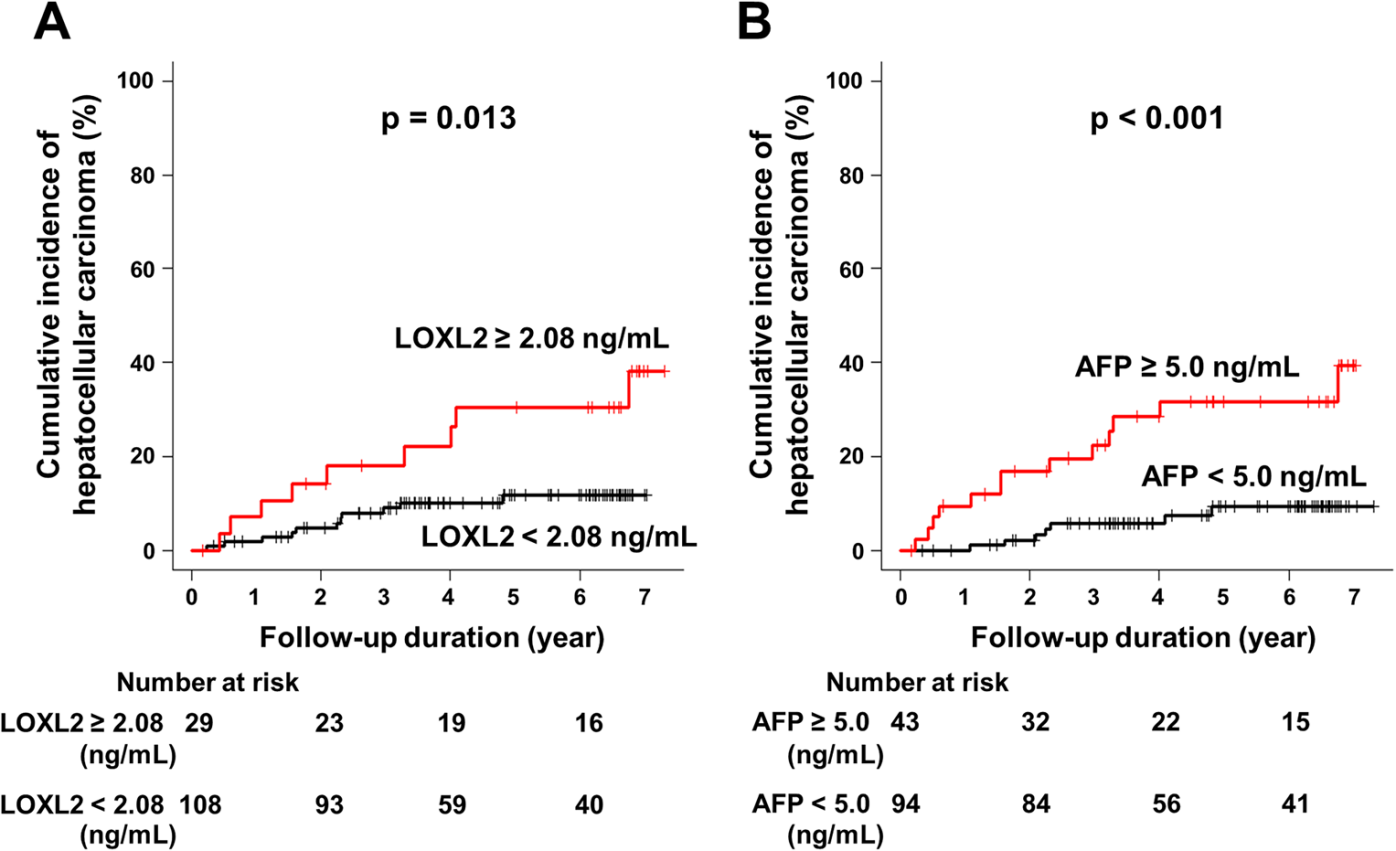
Cumulative Incidence of HCC in Patients who Achieved SVR Stratified according to Serum LOXL2 and AFP Levels. The cumulative post-SVR incidence of HCC development stratified by (**A**) post-Tx serum LOXL2 levels and (B) post-Tx serum AFP levels was assessed using the Kaplan–Meier method. (**A**) The red and black lines indicate post-Tx LOXL2 levels stratified by ≥ 2.08 and < 2.08 ng/mL, respectively. The incidence rate in patients with LOXL2 levels ≥ 2.08 ng/mL was significantly higher than that in patients with lower LOXL2 levels (*p* = 0.013, log-rank test). (**B**) The red and black lines indicate post-Tx AFP levels stratified by ≥ 5.0 and < 5.0 ng/mL, respectively. The incidence rate in patients with AFP levels ≥ 5.0 ng/mL was significantly higher than that in patients with lower AFP levels (p < 0.001, log-rank test). Abbreviations: AFP, alpha-fetoprotein; HCC, hepatocellular carcinoma; LOXL2, lysyl oxidase-like 2; post-Tx, post-treatment; SVR, sustained virological response.

In the comparison of patients with post-Tx LOXL2 levels < 2.08 versus  ≥ 2.08 ng/mL, the cumulative incidence rates of HCC were 1.9% versus 7.1% at 1 year, 9.1% versus 18.2% at 3 years, and 11.9% versus 30.5% at 5 years. When comparing patients with post-Tx AFP levels < 5.0 versus  ≥ 5.0 ng/mL, the cumulative incidence rates of HCC were 0% versus 9.5% at 1 year, 5.8% versus 22.4% at 3 years, and 9.3% versus 31.7% at 5 years, respectively (Fig. 2A,B).

Stratifications by post-Tx levels of platelet count (< 120 vs. ≥ 120 × 10^9^/L), WFA^+^-M2BP (< 1.0 vs. ≥ 1.0 COI), and FIB-4 index (< 2.9 vs. ≥ 2.9) also revealed significant difference in the post-SVR incidence rate of HCC, while no significant difference was observed with ALBI (< -2.27 vs. ≥ -2.27) (Supplementary Fig. 4).

**Figure 3:**
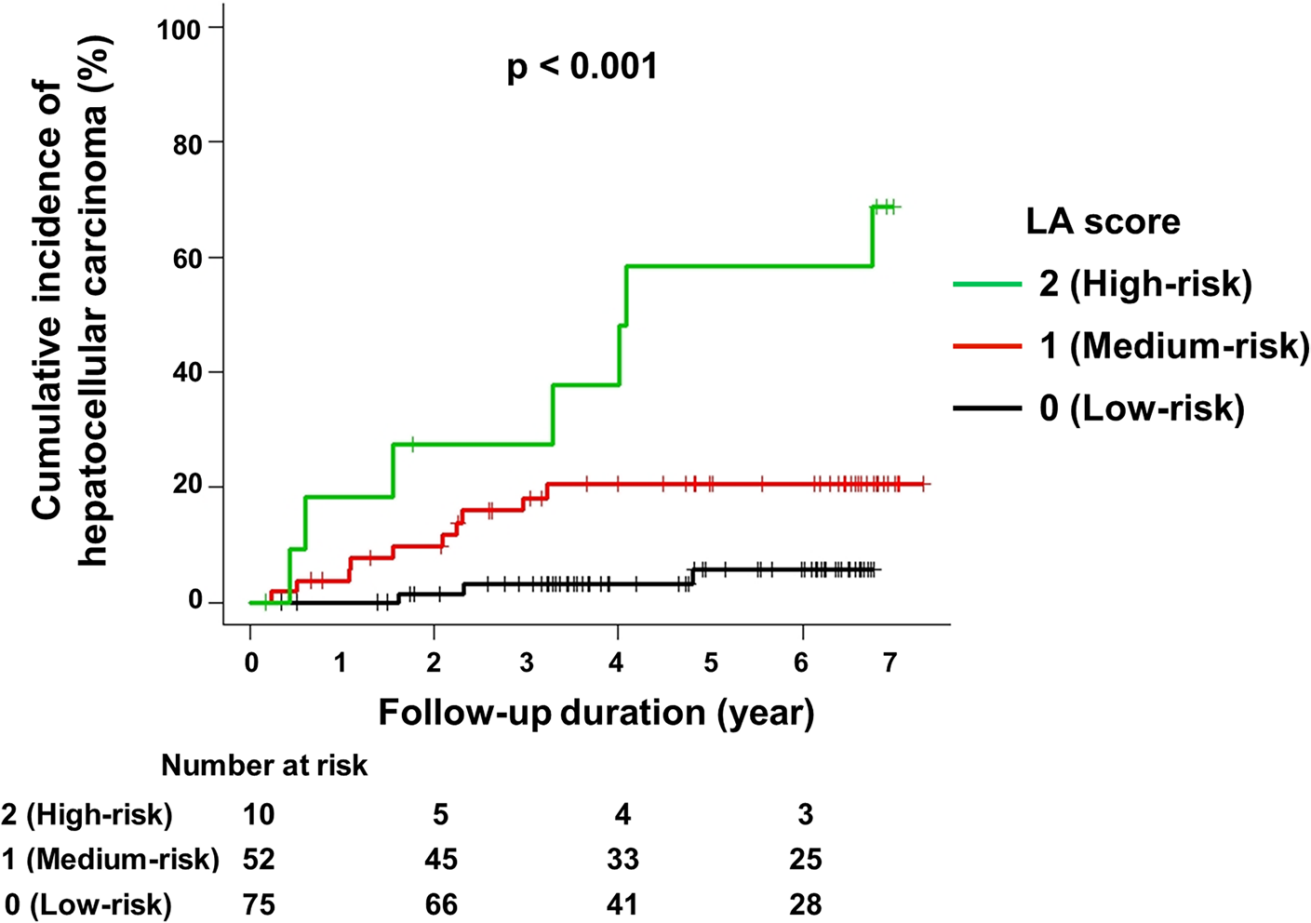
Stratification for the Risk of HCC Development after SVR by Combining LOXL2 and AFP Levels. The cumulative post-SVR incidence of HCC development was significantly stratified (*p* < 0.001) by the LA score. The green, red, and black lines represent the 2-point group in the LA scoring system (high-risk), the 1-point group (medium-risk), and the 0-point group (low-risk), respectively. The cumulative post-SVR incidence of HCC development was assessed using the Kaplan–Meier method and log-rank test. Abbreviations: AFP, alpha-fetoprotein; HCC, hepatocellular carcinoma; LA score, LOXL2-AFP score; LOXL2, lysyl oxidase-like2; SVR, sustained virological response.

To stratify patients for risk of post-SVR HCC development, we developed a scoring system using post-Tx LOXL2 and AFP levels (LOXL2-AFP or LA score). The number of risk factors was counted and directly used for risk stratification. Ten patients (7.3%) achieved 2 points in the LA scoring system (high-risk group; levels of LOXL2 ≥ 2.08 ng/mL and of AFP ≥ 5.0 ng/mL); 52 patients (38.0%) 1 point (medium-risk group; either LOXL2 levels ≥ 2.08 ng/mL or AFP levels ≥ 5.0 ng/mL); and the remaining 75 patients (54.7%) scored 0 points (low-risk group; levels of LOXL2 < 2.08 ng/mL and of AFP < 5.0 ng/mL) (Fig. 3). Based on the LA system, the cumulative post-SVR incidence of HCC was highest for the 2-point group, next for the 1-point group, and minimum for the 0-point group (*p* < 0.001). The cumulative post-SVR incidence of HCC at 1, 3, and 5 years was 22.2%, 33.3%, and 60.0% for the 2-point group; 3.8%, 16.1%, and 20.9% for the 1-point group; and 0%, 4.5%, and 7.1% for the 0-point group, respectively.

## Discussion

The present study demonstrates the utility of serum LOXL2 levels in predicting HCC development after achieving SVR. Given that liver fibrosis is one of the significant factors in post-SVR HCC development, various markers that reflect the severity of fibrosis, such as the FIB-4 index, WFA^+^‐M2BP levels, and platelet counts, have been extensively investigated and reported as predictors^7,34,36–39,43^. Degradation of the deposited fibers in the ECM occurs in patients who have achieved SVR following DAA therapy^25–27^. The wide variation in the rate and extent of fiber degradation among patients who achieve SVR implies that fiber insolubility or stability affects post-SVR HCC development. Therefore, we specifically focused on the association between post-translational fiber modifications and post-SVR HCC development, and then validated the effectiveness of the novel parameter LOXL2.

In our initial investigation, we characterized the serum LOXL2 levels as a clinical marker. The concentration of serum LOXL2 was elevated in patients infected with HCV compared to that in healthy individuals, and it rapidly decreased following HCV clearance, suggesting a parallel with the resolution of inflammation. This pattern was also observed for the FIB-4 index and WFA^+^-M2BP levels, which decreased significantly after SVR was achieved. LOXL2 is induced via various pathways. Notably, the promoter region of LOXL2 contains the SMAD binding sequence, indicating that LOXL2 expression increases in parallel with virus-induced inflammation and subsequent TGF-β expression^17^. LOXL2 expression is also triggered by the hypoxia/hypoxia-inducible factor-1 (HIF-1) pathway, or by ECM stiffness followed by the activation of integrins, c-Jun N-terminal kinase (JNK)/c-Jun, or MEK1/2-ERK1/2^17,44^.

Besides its diverse induction pathways, LOXL2 exhibits a variety of functions other than ECM cross-linking, including EMT regulation, cell migration, and angiogenesis^16–21^. Surprisingly, two forms of LOXL2 have been described: one secreted into the extracellular space, and the other, which features a modified N-terminus, retained within cells. The function of the latter has not been fully elucidated, but it appears to perturb tumor-related pathways and possibly regulate cell death or proliferation within hepatocytes^19,45–47^. Considering its diverse induction pathways and functions, it is more reasonable to regard LOXL2 levels as a pathophysiological biomarker along a novel axis, rather than as a marker in the context of liver fibrosis.

We demonstrated that serum post-Tx LOXL2 levels ≥ 2.08 ng/mL is an independent factor for predicting HCC development after achieving SVR. We also confirmed that known fibrosis markers at the pre-Tx period (platelet count < 120 × 10^9^/L, AFP levels ≥ 10.0 ng/mL, FIB-4 index ≥ 3.25, WFA^+^-M2BP ≥ 2.8 COI) or post-Tx (platelet count < 120 × 10^9^/L, AFP ≥ 5.0 ng/mL, FIB-4 index ≥ 2.9, WFA^+^-M2BP ≥ 1.0 COI) were significant. Although it has not been conclusively determined whether pre- or post-Tx assessment is more advantageous, our results indicated that post-Tx assessment tended to have higher HRs in the univariate analysis than pre-Tx, except for the FIB-4 index. Sato et al. reported similar results regarding WFA^+^-M2BP levels, supporting the notion that post-Tx evaluation is a more robust predictive factor for post-SVR HCC development^43^. In predicting HCC development after achieving SVR, it might be crucial to evaluate post-Tx risk factors that are not affected by inflammation.

Serum LOXL2 levels between the patients with HCC and those without HCC were not statistically different in Table 2; however, when utilizing the most suitable cut-off value calculated from the ROC curve for predicting HCC, serum LOXL2 level was found to be a significant predictive factor (Table3). The reason for this discrepancy is considered to be that the mean value was influenced by some cases, especially in the patients without HCC, that deviate from the mean.

Lastly, we identified high serum levels of LOXL2 (≥ 2.08 ng/mL) and AFP (≥ 5.0 ng/mL) as independent risk factors of HCC development after achieving SVR. The underlying mechanism for post-SVR HCC development is multifactorial and includes persistent metabolic abnormalities after virus clearance, damage resulting from other liver diseases, and severe liver fibrosis without degradation^48,49^. Therefore, predictions using a single marker that reflects a single pathophysiological characteristic are challenging. We stratified patients to further differentiate the risk using serum levels of LOXL2 ≥ 2.08 ng/mL and of AFP ≥ 5.0 ng/mL. We established the LA score, which comprises post-Tx serum LOXL2 and AFP levels, and validated performance in our cohort. The LA score identified three groups significantly different regarding the risk of HCC development after achieving SVR: a high-risk 2-point group (with levels of LOXL2 ≥ 2.08 ng/mL and of AFP ≥ 5.0 ng/mL), a medium-risk 1-point group (levels of either LOXL2 ≥ 2.08 ng/mL or of AFP ≥ 5.0 ng/mL), and a low-risk 0-point group (lower LOXL2 and AFP levels). Previous studies have also succeeded in improving the accuracy of HCC prediction after achieving SVR by combining multiple markers^50–52^.

Interestingly, the combinations of serum LOXL2 levels ≥ 2.08 ng/mL with a platelet count < 120 × 10^9^/L, age ≥ 65 years, or a diagnosis with diabetes mellitus were identified as independent risk factors in the multivariate analysis, but effective risk stratification was not achieved. Furthermore, when the serum LOXL2 level was combined with the FIB-4 index or WFA^+^-M2BP level, only LOXL2 was extracted as a predictive factor. As expected, stratified analysis using the combination of LOXL2 level and FIB-4 index or WFA^+^-M2BP level did not achieve effective risk stratification (data not shown). The level of serum AFP is an excellent predictor of HCC development after achieving SVR, particularly in terms of specificity rather than sensitivity^31,53^. Rocha et al. have reported the efficacy of this parameter in predicting HCC after SVR, suggesting that further reinforcement is necessary in conjunction with other markers^53^. In the current results, serum LOXL2 levels showed superior sensitivity. One reason why consideration of serum AFP levels with LOXL2 levels allows for effective stratification appears to be that the serum AFP and LOXL2 levels may mutually complement their weaknesses.

In recent clinical trials, simtuzumab, a monoclonal antibody against LOXL2, failed to effectively resolve fibrosis in patients with non-alcoholic steatohepatitis (NASH)^54^. This negative outcome could be related to an inefficient antibody product rather than LOXL2 not being a relevant target for antifibrotic therapy^55^. Since LOXL2 is a multifunctional molecule associated with various biological processes, efforts to target it for diagnosis or treatment are still ongoing^20,55–57^.

The present study has certain limitations. First, the lack of a validation cohort made it impossible to assess the reproducibility of the results. Second, a small number of patients even in the 0-point group based on the LA score developed post-SVR HCC, indicating that the proposed system did not identify individuals at zero risk of developing the disease. This issue is crucial, because it is clinically significant to identify patients with no risk of developing post-SVR HCC and thus require no further surveillance. Addressing this limitation requires further investigation. Lastly, the retrospective design and potential selection bias in our study should not be neglected. Given the limited number of patients with HCC who achieved SVR, we were unable to use Cox proportional hazards analysis with multiple factors to extract the strongest predictors. Regarding metabolic disorders, our study confirmed that a pre-Tx diagnosis with diabetes mellitus was an independent risk factor along with serum LOXL2 levels; however, the number of patients who were positive for both parameters was too small (N = 5) for evaluation. Metabolic disorders have been actively investigated as factors associated with HCC development after achieving SVR and promising results have been reported^58–60^. The use of metabolic factors in predicting post-SVR HCC development remains to be addressed. Our cohort exhibited potential biases due to the restriction of cases to genotype 1 and a higher proportion of cases with advanced fibrosis. The latter was attributed to the approval of daclatasvir (DCV)/asunaprevir (ASV) in 2014 as the first interferon-free treatment in Japan, which was used for many cases with advanced fibrosis ineligible for interferon therapy. Furthermore, our emphasis on managing high-risk cases with advanced fibrosis may have led to the loss of follow-up for some cases with relatively mild fibrosis after achieving SVR. It is speculated that these biases may account for the increased incidence of HCC development after achieving SVR in our cohort compared to recent reports^8,61^.

To address these issues, more cases are necessary to conduct a larger-scale prospective study.

## Conclusions

This study identified post-Tx serum LOXL2 levels as a valuable predictive marker for HCC development after achieving SVR. Additionally, a new scoring system that combines post-Tx serum LOXL2 and AFP levels allowed stratification of patients according to their risk of post-SVR HCC incidence. Future prospective studies with larger cohorts are required to validate our predictive model for HCC development after achieving SVR.

## Methods

### Patients

This retrospective multicenter cohort study conducted by the DAA Study in Hamamatsu group (DASH study group), involved 9 institutions (Hamamatsu University Hospital, Shimada General Medical Center, Seirei Hamamatsu General Hospital, Hamamatsu Medical Center, Iwata City Hospital, Shizuoka City Shizuoka Hospital, Minoru Medical Clinic, Tamakoshi Clinic, Elm Medical Clinic). The study was approved by the Ethics Committee of Hamamatsu University School of Medicine (approval number, 23-224). The study protocols conformed to the ethical guidelines of the Declaration of Helsinki. All patients provided written informed consent. Patients diagnosed with serogroup 1 chronic HCV infection who initiated IFN-free DAA regimens (DCV/ASV or sofosbuvir [SOF]/ledipasvir [LDV]) between August 2014 and December 2016 at nine affiliated institutions were enrolled. SVR was defined as absence of detectable HCV RNA at 24 weeks after treatment completion. No relapse of viremia was observed after 24 weeks in the patients who achieved SVR. Serum samples from healthy volunteers were also collected during the study period.

**Figure 4:**
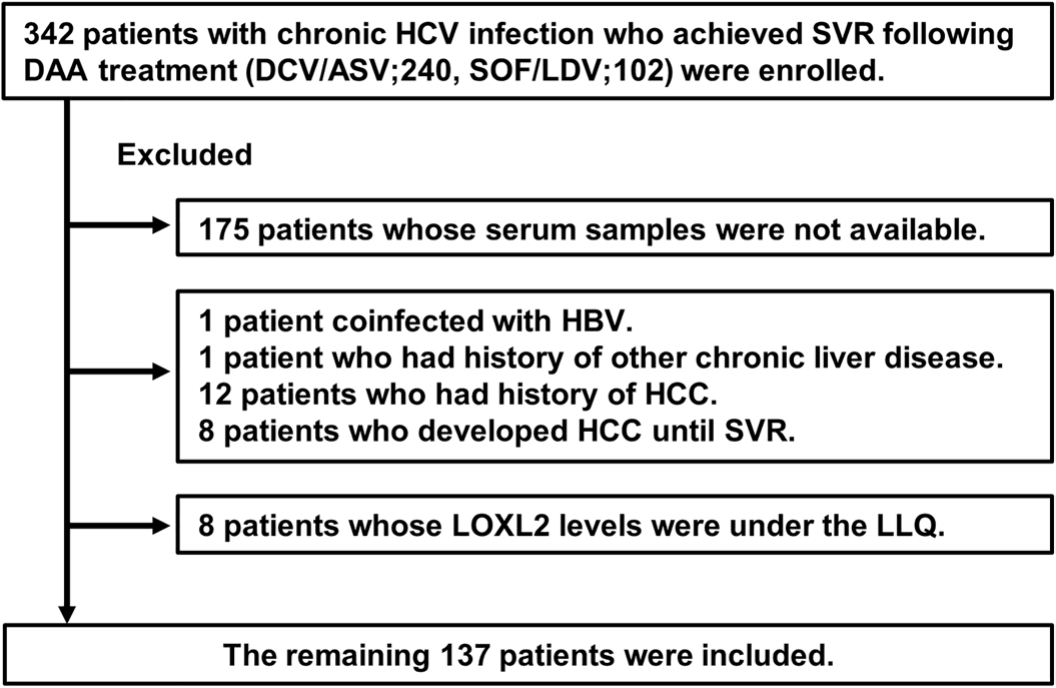
Study Flowchart Illustrating the Patient Selection Process. The development of HCC was studied in patients with HCV who achieved SVR via DAA therapy. Abbreviations: ASV, asunaprevir; DAA, direct-acting antiviral; DCV, daclatasvir; HBV, hepatitis B virus; HCC, hepatocellular carcinoma; HCV, hepatitis C virus; LDV, ledipasvir; LLQ, lower limit of quantification; LOXL2, lysyl oxidase-like 2; SOF, sofosbuvir; SVR, sustained virological response.

The exclusion criteria were as follows: (1) absence of sufficiently stored serum samples; (2) coinfection with either hepatitis B virus (HBV) or human immunodeficiency virus; (3) history of other chronic liver diseases (autoimmune hepatitis, primary biliary cholangitis, hemochromatosis, or Wilson’s disease); (4) history of HCC development at enrollment; (5) HCC development in the period up to 24 weeks after treatment completion, detected using ultrasonography (US), contrast-enhanced computed tomography (CT), or contrast-enhanced magnetic resonance imaging (MRI); (6) serum LOXL2 levels (see below) under the lower limit of quantification (LLQ). After exclusions, the data from 137 patients were retrospectively analyzed to identify risk factors for HCC development after achieving SVR (Fig. 4).

### Laboratory tests

Blood samples were collected on the day before the initiation of DAA treatment (pre-Tx) and 24 weeks after treatment completion (post-Tx). Serum samples were separated and stored at − 80 °C. Hematological and biochemical parameters, including platelet counts, levels of AST, ALT, total bilirubin, gamma-glutamyl transpeptidase (GGT), albumin, and AFP, and HCV serogroup were measured at each institution using standard techniques. Measurement of the WFA^+^-M2BP levels were performed by the contract laboratory (SRL Inc, Tokyo, Japan) using stored serum. Serum HCV-RNA titers were measured using the Roche COBAS TaqMan test (LLQ; 1.2 log_10_ IU/mL; Roche Molecular Diagnostics, CA) or AccuGene m-HCV (LLQ; 1.1 log_10_ IU/mL; Abbott Japan, Tokyo, Japan). The FIB-4 index and ALBI scores were calculated and used as surrogate indicators of liver fibrosis and function, respectively^62,63^.

### Measurement of human LOXL2 levels

Stored pre-Tx and post-Tx serum samples were used to quantify LOXL2 levels using an enzyme-linked immunosorbent assay (ELISA) kit (Cloud-Clone Corp, TX). The detection range was 0.156–10 ng/mL.

### Surveillance and diagnosis of HCC

All patients were followed-up at intervals of 1–6 months by measuring their biochemical and virological values and blood counts. Imaging examinations (US, contrast-enhanced CT, or contrast-enhanced MRI) were performed at least once every 6 months. A diagnosis of HCC development was based on findings of typical vascular patterns on contrast-enhanced CT or MRI.

### Statistical analysis

All statistical analyses were performed using SPSS ver. 25.0 (SPSS, Chicago, IL, USA), GraphPad Prism (ver. 9.0; GraphPad Software, San Diego, CA), and EZR (ver 1.60; Saitama Medical Center, Jichi Medical University, Saitama, Japan). The data are presented as medians and ranges, or numbers and percentages. The chi-square and Mann–Whitney U tests were used to evaluate categorical data and continuous variables, respectively. The Wilcoxon signed-rank test was used to assess differences between the two paired groups. The Cox proportional hazards model was used to evaluate the association between each variable and HCC development (univariate and multivariate analyses). The Kaplan–Meier method and log-rank test were used to assess the cumulative incidence of HCC in each group. The correlation between the two variables was evaluated using Pearson’s correlation coefficient. p < 0.05 was considered to indicate statistical significance.

### Supplementary Information

Supplementary Information.

## Data Availability

The datasets generated and/or analyzed during this study are available from the corresponding author on reasonable request.

## Abbreviations

AFP: Alpha-fetoprotein
ALBI: Albumin-bilirubin
ALT: Alanine aminotransferase
ANOVA: Analysis of variance
ASV: Asunaprevir
AST: Aspartate aminotransferase
BMI: Body mass index
CI: Confidence interval
COI: Cut-off index
CT: Computed tomography
DAA: Direct-acting antiviral
DCV: Daclatasvir
ECM: Extracellular matrix
ELISA: Enzyme-linked immunosorbent assay
EMT: Epithelial-mesenchymal transition
ERK1/2: Extracellular signal-regulated kinase 1/2
FIB-4: Fibrosis-4
GGT: Gamma-glutamyl transpeptidase
HIF-1: Hypoxia-inducible factor-1
HBV: Hepatitis B virus
HCC: Hepatocellular carcinoma
HCV: Hepatitis C virus
HR: Hazard ratio
IFN: Interferon
Jnk: C-jun N-terminal kinase
LA score: LOXL2-AFP score
LDV: Ledipasvir
LLQ: Lower limit of quantification
LOX: Lysyl oxidase
LOXL2: Lysyl oxidase-like 2
MEK1/2: Mitogen-activated protein kinase 1/2
MRI: Magnetic resonance imaging
NASH: Non-alcoholic steatohepatitis
pre-Tx: Pre-treatment
post-Tx: Post-treatment
ROC: Receiver operating characteristic
SOF: Sofosbuvir
SVR: Sustained virological response
US: Ultrasonography
WFA^+^-M2BP: Wisteria floribunda agglutinin-positive human Mac-2 binding protein
