# Supplementary material for: Lysyl oxidase-like 2 as a predictor of hepatocellular carcinoma in patients with hepatitis C virus after sustained virological response
Source: Sci Rep. 2024 May 13;14:10864. doi: 10.1038/s41598-024-61366-y (PMC11091085; doi:10.1038/s41598-024-61366-y)
Supplement: Supplementary file 1 — Supplementary Information. [file 41598_2024_61366_MOESM1_ESM.pdf]

Supplementary Information

Lysyl oxidase-like 2 as a predictor of hepatocellular carcinoma in patients with hepatitis C virus after sustained virological response

Takeshi Chida*[1 2], Kazuyoshi Ohta[1], Hidenao Noritake[1], Masahiro Matsushita[3], Gou Murohisa[4], Fujito Kageyama[5], Yuzo Sasada[6], Tatsuki Oyaizu[7], Minoru Tsugiki[8], Katsutoshi Tamakoshi[9], Takeyuki Nakajima[10], Takafumi Suda[1], Kazuhito Kawata[1]

[1] Second Department of Internal Medicine, Hamamatsu University School of Medicine, 1-20-1 Handayama, Hamamatsu, Shizuoka 431-3192, Japan
[2] Department of Regional Medical Care Support, Hamamatsu University School of Medicine, 1-20-1 Handayama, Hamamatsu, Shizuoka 431-3192, Japan
[3] Department of Gastroenterology, Shimada General Medical Center, 1200-5 Noda, Shimada, Shizuoka 427-8502, Japan
[4] Department of Hepatology, Seirei Hamamatsu General Hospital, 2-12-12 Sumiyoshi, Hamamatsu, Shizuoka 430-8558, Japan
[5] Department of Hepatology, Hamamatsu Medical Center, 328 Tomitsuka-cho, Hamamatsu, Shizuoka 432-8580, Japan
[6] Department of Hepatology, Iwata City Hospital, 512-3 Ookubo, Iwata, Shizuoka 438-8550, Japan
[7] Department of Gastroenterology, Shizuoka City Shizuoka Hospital, 10-93 Otemachi, Shizuoka, Shizuoka 420-8630, Japan
[8] Minoru Medical Clinic, 1784-1 Mishima-cho, Hamamatsu, Shizuoka 430-0853, Japan
[9] Tamakoshi Clinic, 262-1 Maruzuka-cho, Hamamatsu, Shizuoka 435-0046, Japan
[10] Elm Medical Clinic, 5-17-22 Handayama, Hamamatsu, Shizuoka 431-3125, Japan

Supplementary Figure 1

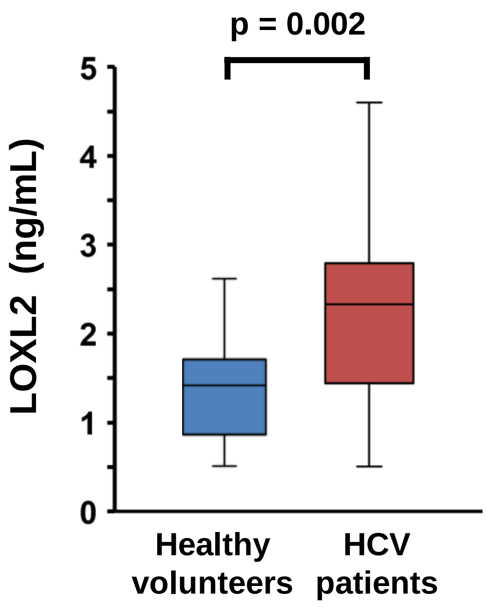

Supplementary Figure 1. Comparison of LOXL2 Levels in Healthy Volunteers with Those in Patients Infected with HCV. Statistical analysis was performed using the Mann–Whitney U test. Abbreviations: HCV, hepatitis C virus; LOXL2, lysyl oxidase-like 2.

Supplementary Figure 2

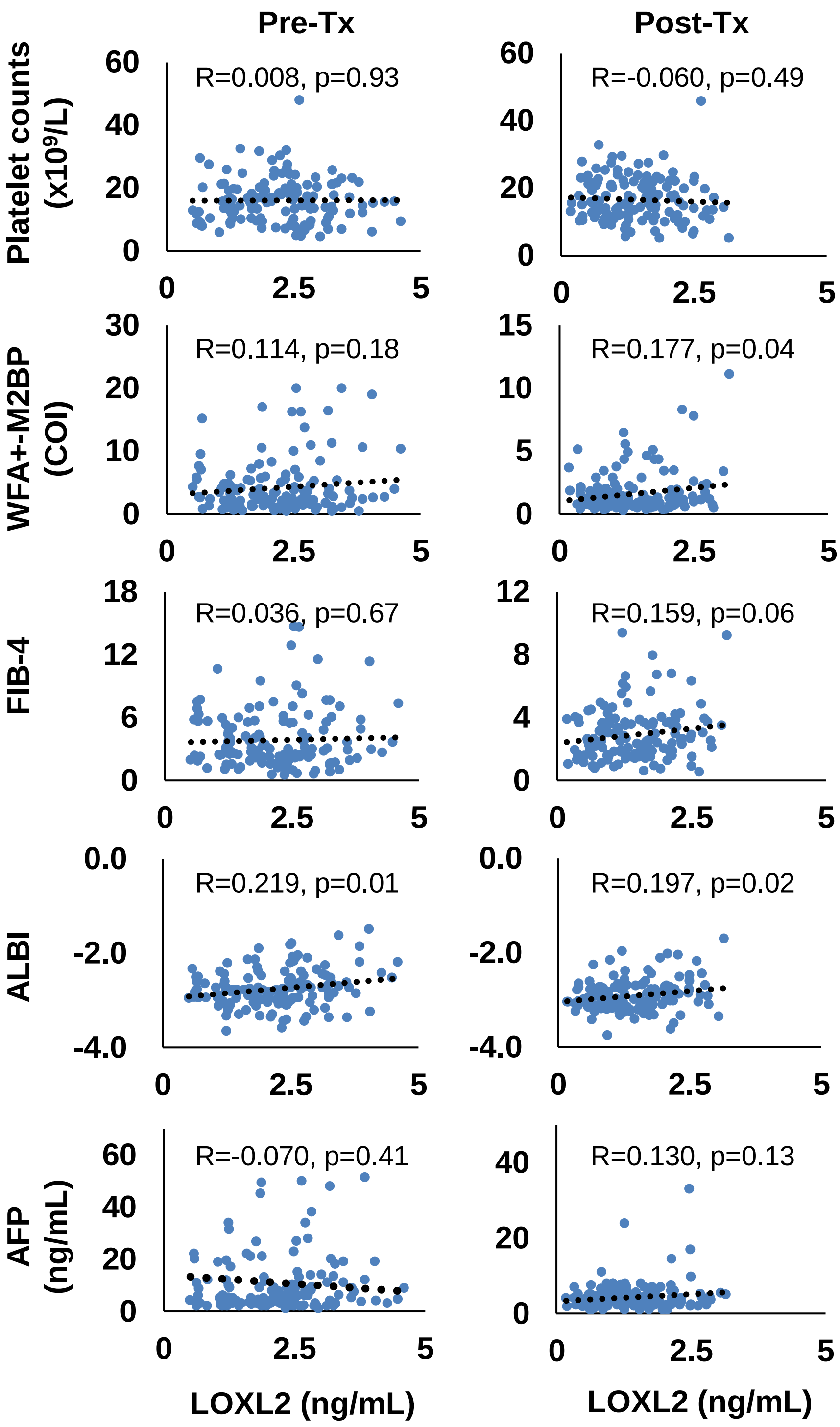

Supplementary Figure 2. Correlation between LOXL2 Levels and Liver Fibrosis Markers or AFP Levels. Pearson's correlation coefficients were used for the analyses. Abbreviations: AFP, alpha-fetoprotein; COI, cut off index; FIB - 4, fibrosis - 4; LOXL2, lysyl oxidase-like 2; pre-Tx, pre-treatment; post-Tx, post-treatment; WFA^{+}-M2BP, Wisteria floribunda agglutinin-positive human Mac-2 binding protein.

Supplementary Figure 3

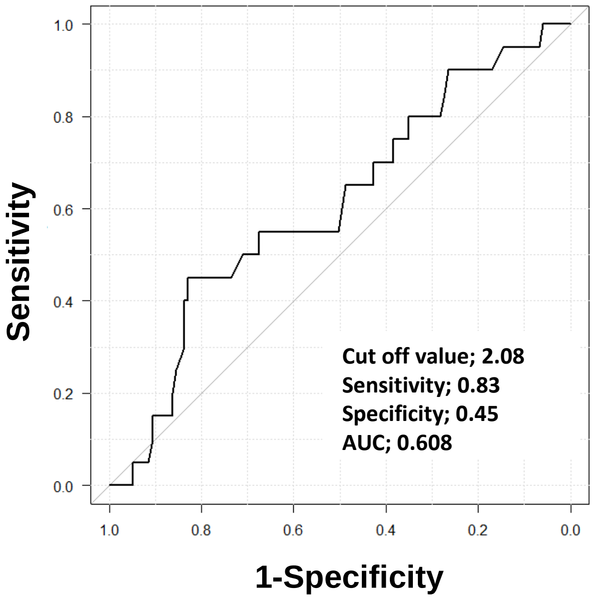

Supplementary Figure 3. ROC Curve of post-Tx LOXL2 Levels for Prediction of HCC Development after SVR. The area under the curve (AUC) value for the HCC after SVR is 0.608. Abbreviations: AUC, area under the curve; HCC, hepatocellular carcinoma; LOXL2, lysyl oxidase-like 2; post-Tx, post-treatment; ROC, receiver operating characteristic; SVR, sustained virological response.

Supplementary Figure 4

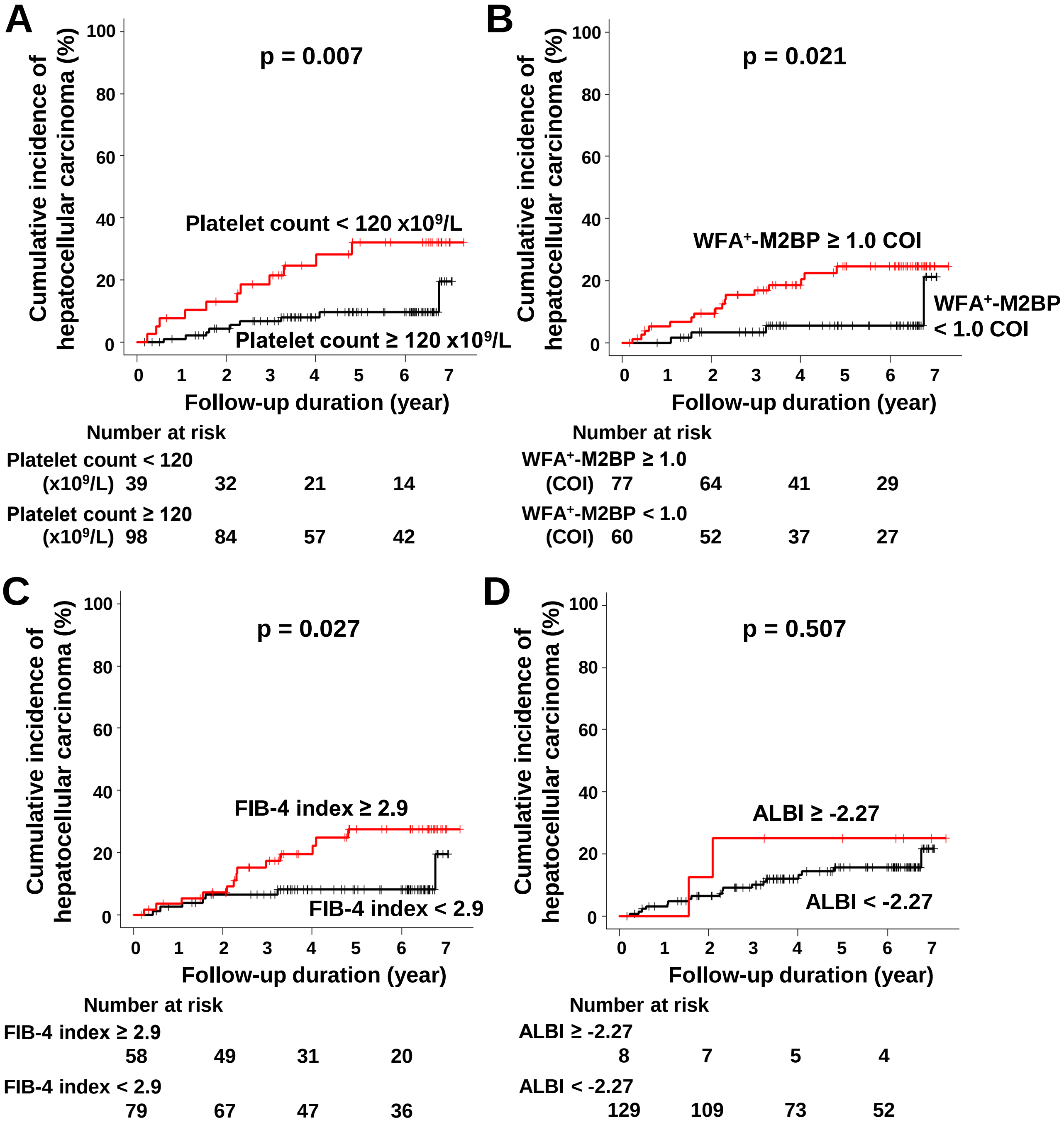

Supplementary Figure 4. Cumulative Incidence of HCC in Patients who Achieved SVR Stratified according to risk factors.

The cumulative post-SVR incidence of HCC development stratified by (A) post-Tx platelet count, (B) post-Tx WFA$^+$-M2BP levels, (C) post-Tx FIB-4 index, and (D) post-Tx ALBI was assessed using the Kaplan–Meier method and log-rank test. The red and black lines indicate high risk group and low risk group, respectively. Abbreviations: ALBI, albumin–bilirubin; COI, cut off index; FIB-4, fibrosis-4; HCC, hepatocellular carcinoma; post-Tx, post-treatment; SVR, sustained virological response; WFA$^+$–M2BP, Wisteria floribunda agglutinin-positive human Mac-2 binding protein.

Supplementary Table 1

Case #	1	2	3	4	5	6	7	8	9	10	11	12	13	14	15	16	17	18	19	20
Age (year)	48	69	74	79	78	82	70	65	77	43	68	82	65	78	70	65	72	72	66	76
Sex (M:male, F:female)	M	M	F	M	F	M	M	M	F	M	M	F	M	F	F	M	M	F	M	F
BMI (kg/m^2)	31.2	24.4	25.3	20.7	20.4	21.2	20.8	25.2	22.7	31.6	21.5	20.1	22.9	23.4	26.7	16.8	21.5	20.5	25.0	20.8
Alcohol intake ≥20g/day	N	Y	N	N	N	N	N	N	N	N	N	N	N	N	N	N	N	N	N	N
Diabetes mellitus	Y	N	N	N	N	N	Y	N	N	Y	Y	N	Y	N	N	N	Y	N	N	N
Past history of IFN treatment	Y	Y	Y	Y	N	N	Y	Y	N	Y	Y	N	N	Y	Y	Y	N	N	N	N
DAA regimen	S/L	S/L	S/L	D/A	D/A	D/A	D/A	D/A	D/A	D/A	D/A	D/A	S/L	S/L	D/A	D/A	D/A	D/A	D/A	D/A
Pre-Tx HCV titer (Log$_{10}$ IU/mL)	5.7	5.0	6.9	5.5	6.6	6.2	6.4	5.0	5.6	6.2	5.9	3.2	5.1	5.7	6.7	5.0	5.5	5.2	5.6	6.0
Observation period (year)	0.4	3.2	4.8	1.6	3.3	2.1	4.0	1.6	2.3	1.6	0.2	0.5	1.1	2.2	6.8	1.1	2.3	4.1	0.6	3.0
Platelet counts (x10^9/L)																				
At Pre-Tx	66	153	107	173	82	144	89	124	164	135	59	105	192	97	201	71	119	171	186	105
At Post-Tx	65	166	110	211	109	172	110	110	141	221	70	107	188	72	180	99	105	198	221	119
AST (U/L)																				
At Pre-Tx	39	54	36	25	38	63	30	49	37	57	124	29	125	39	33	41	146	44	52	81
At Post-Tx	25	19	27	19	21	31	16	34	24	22	34	32	21	29	20	21	26	36	26	25
ALT (U/L)																				
At Pre-Tx	55	69	20	21	19	54	24	45	31	68	180	27	160	25	28	37	233	26	56	72
At Post-Tx	50	12	15	14	5	16	12	27	13	24	32	14	17	16	11	13	21	19	18	17
Total bilirubin (mg/dL)																				
At Pre-Tx	0.4	0.5	1.4	0.6	0.6	0.7	0.8	1.1	0.7	0.3	1.1	1.5	0.6	0.9	0.5	0.6	1.0	1.6	0.4	0.8
At Post-Tx	0.7	0.6	1.2	1.3	0.4	0.9	0.4	1.8	0.6	0.3	1.2	1.9	0.4	0.7	0.5	0.8	0.8	1.1	0.3	0.6
GGT (U/L)																				
At Pre-Tx	37	66	27	22	24	21	35	37	38	92	87	18	64	23	38	24	25	26	57	55
At Post-Tx	29	14	39	23	13	12	18	28	26	33	40	25	28	20	27	19	19	48	34	19
Albumin (g/dL)																				
At Pre-Tx	3.9	3.9	4.8	4.0	3.6	3.0	4.1	3.6	3.7	3.7	4.2	3.8	3.7	3.4	4.2	5.0	3.7	4.2	4.0	3.4
At Post-Tx	4.2	4.0	4.6	4.3	4.2	3.3	4.2	3.7	4.1	3.8	4.8	4.2	4.2	4.1	4.0	5.0	4.0	4.4	3.9	4.0
AFP (ng/mL)																				
At Pre-Tx	8.0	8.0	4.0	4.0	34.0	12.0	11.0	9.0	19.4	38.1	18.9	45.2	11.4	9.3	15.0	5.1	22.1	5.2	5.8	21.0
At Post-Tx	33.0	5.0	4.0	3.0	6.0	2.0	6.0	5.0	11.0	17.0	7.9	23.9	5.3	4.8	7.5	3.2	3.9	4.4	14.4	6.6
WFA$^+$-M2BP (COI)																				
At Pre-Tx	3.35	1.49	3.16	3.77	13.8	2.33	1.70	4.22	1.40	4.53	3.44	5.64	5.90	10.4	1.95	5.48	5.73	3.65	2.77	7.17
At Post-Tx	1.39	0.39	1.99	1.08	3.47	1.90	1.01	2.88	1.19	0.98	1.00	4.92	0.85	4.32	0.89	1.55	2.00	1.73	1.48	1.97
FIB-4 index																				
At Pre-Tx	3.82	2.93	5.57	2.49	8.29	4.88	4.82	3.83	3.12	2.20	10.7	4.36	3.35	6.27	2.17	6.17	5.79	3.63	2.47	6.91
At Post-Tx	2.67	2.31	4.75	1.93	6.81	3.74	2.98	3.93	3.68	0.89	5.92	6.63	1.79	7.95	2.38	3.88	3.94	3.04	1.86	3.92
ALBI																				
At Pre-Tx	-2.76	-2.70	-3.17	-2.73	-2.39	-1.86	-2.74	-2.22	-2.45	-2.68	-2.73	-2.30	-2.48	-2.11	-2.95	-3.58	-2.33	-2.62	-2.85	-2.14
At Post-Tx	-2.86	-2.73	-3.04	-2.77	-3.02	-2.02	-3.03	-2.16	-2.84	-2.76	-3.21	-2.57	-3.02	-2.77	-2.78	-3.50	-2.65	-2.90	-2.85	-2.73
LOXL2 (ng/mL)																				
At Pre-Tx	2.71	2.82	3.18	2.50	2.71	3.86	3.13	1.26	1.20	2.83	1.04	1.85	1.94	2.83	2.56	2.33	0.58	3.60	2.36	1.66
At Post-Tx	2.48	0.65	0.87	1.19	2.13	2.08	2.19	0.99	0.84	2.49	1.28	1.27	1.71	1.77	2.13	2.21	0.40	2.71	2.14	1.08

Supplementary Table 1. Characteristics of the Patients with HCC Development after Achieving SVR in the Present Study.

Data were presented as numbers and Y/N which indicates Yes or No, respectively. Regarding the DAA regimen, D/A and S/L indicates DCA/ASV and SOF/LDV, respectively. Abbreviations: AFP, alpha-fetoprotein; ALBI, albumin–bilirubin; ALT, alanine aminotransferase; AST, aspartate aminotransferase; ASV, asunaprevir; BMI, body mass index; COI, cut off index; DAA, direct-acting antiviral; DCV, daclatasvir; FIB - 4, fibrosis - 4; GGT, gamma-glutamyl transpeptidase; HCV, hepatitis C virus; IFN, interferon; LDV, ledipasvir; LOXL2, lysyl oxidase-like 2; pre-Tx, pre-treatment; post-Tx, post-treatment; SOF, sofosbuvir; WFA$^+$-M2BP, Wisteria floribunda agglutinin-positive human Mac-2 binding protein.
